# Comparative ChIP-Seq Analysis of ERα Binding Induced by Estradiol, Zearalenone, and Bisphenol A in Human Ovarian Cells

**DOI:** 10.3390/biom16101392

**Published:** 2026-09-24

**Authors:** Lajos Széles, Éva Márton, Bálint Takács, Dóra Domoszlai, Eszter Homoki, Loránd Göczi, András Penyige, István Balogh, Melinda Szilágyi

**Affiliations:** 1 Department of Medical Genetics, Faculty of Medicine, University of Debrecen, 4032 Debrecen, Hungary; szelesl@med.unideb.hu (L.S.); marton.eva@med.unideb.hu (É.M.); domoszlai.dora@med.unideb.hu (D.D.); homoki.eszter@med.unideb.hu (E.H.); goczi.lorand@med.unideb.hu (L.G.); penyige@med.unideb.hu (A.P.); balogh@med.unideb.hu (I.B.); 2 Center for Clinical Genetics, Faculty of Medicine, University of Debrecen, 4032 Debrecen, Hungary; takacs.balint@med.unideb.hu

**Keywords:** estrogen receptor, functional genomics, ChIP-seq, xenoestrogen, endocrine disruptor, zearalenone, bisphenol A, estradiol

## Abstract

Xenoestrogens are synthetic or natural compounds that mimic the effect of endogenous estrogens by binding to estrogen receptors (ERs). Human exposure to xenoestrogens is increasing globally, raising substantial concerns regarding their endocrine-disrupting effects. Here, we investigated the ERα-mediated genomic and transcriptional response of human ovarian cells following exposure to estradiol (E2), zearalenone (ZEA), and bisphenol A (BPA) using ChIP-seq integrated with transcriptomic analysis. Our results demonstrate that the ERα-dependent response induced by ZEA closely resembles that of E2, whereas BPA elicits a markedly attenuated response. ERα signal intensity was higher in cells treated with E2 or ZEA than in cells treated with BPA, indicating reduced ERα occupancy in response to BPA exposure. We identified 641 genomic regions that were occupied by ERα after any treatment, defining a core set of shared binding sites. Integration with mRNA-seq datasets revealed 162 ERα-binding sites located within ±50 kb of the transcription start sites of 109 upregulated genes. Among these, 28 genes were induced across all treatments, constituting a core estrogen-responsive gene set in human ovarian cells. RT-qPCR validation of six representative genes (*GREB1*, *RASGRP1*, *CISH*, *RBBP8*, *KCNF1*, and *LONRF2*) confirmed stronger and sustained transcriptional induction following E2 and ZEA exposure than following BPA exposure; this induction was inhibited by the ERα antagonist MPP. Our findings suggest that BPA has a lower capacity to activate ERα chromatin binding and downstream transcriptional programs, affecting primarily high-affinity estrogen-responsive loci in human ovarian cells.

## 1. Introduction

The term xenoestrogens encompasses both naturally occurring compounds (produced by plants or fungi) and synthetic agents (including industrial chemicals, pharmaceuticals, and personal care products) that mimic the biological activity of endogenous estrogens [1]. Bisphenol A (BPA) is a synthetic xenoestrogen extensively utilized in the manufacture of polycarbonate plastics and epoxy resins, as well as in dental sealants, medical devices, and the internal coatings of food and beverage containers. Human exposure to BPA is considered substantial, largely due to its propensity to leach from plastic items and contaminate food and liquids stored in BPA-containing materials. BPA is readily absorbed through the gastrointestinal tract; however, inhalation and dermal uptake also represent relevant routes of human exposure. Consequently, BPA is readily detectable in human biological fluids, including plasma, urine, and amniotic fluid [2,3]. Zearalenone (ZEA) is a mycotoxin, also referred to as a mycoestrogen, produced by *Fusarium* species, including *F. graminearum*, *F. culmorum*, and *F. oxysporum*. These fungi commonly infect maize and other cereal crops both in the field and during storage. ZEA exhibits high chemical stability, allowing it to persist through food processing and thereby contributing to the frequent contamination of cereal-derived products such as bread, malt, and flour. In addition to direct dietary exposure, humans may also encounter ZEA indirectly through the consumption of animal-derived products, including meat and milk, as livestock are often exposed to mycotoxins via contaminated feed [4]. The effects of xenoestrogens are primarily mediated through binding to estrogen receptors (ERs), particularly ERα, thereby eliciting estrogenic responses. Beyond receptor binding, xenoestrogens disrupt endogenous hormonal signaling through multiple mechanisms, including modulation of hormone receptor expression, perturbation of hormone synthesis or metabolism, and interference with epigenetic regulation [5,6]. Owing to these multifaceted effects on the endocrine system, xenoestrogens are classified as endocrine-disrupting chemicals and have been implicated in a range of pathological conditions, including infertility, precocious puberty, and hormone-dependent cancers [2,5,7,8,9,10]. Beyond their endocrine effects, xenoestrogens may perturb immune homeostasis by modulating proinflammatory cytokine production, thereby increasing the risk of immune-related disorders [11,12].

Estrogen signaling is mediated through multiple mechanisms, including (i) genomic, (ii) tethered, (iii) non-genomic, and (iv) ligand-independent pathways. Among these, genomic signaling is driven by intracellular ERs, namely ERα and ERβ, which function as ligand-activated transcription factors within the nuclear receptor superfamily. Structurally, ERs comprise six functional domains (A–F), including the DNA-binding domain (DBD; C domain) and the ligand-binding domain (LBD; E domain). The DBD specifically recognizes the consensus estrogen response element (ERE) motif (GGTCAnnnTGACC), whereas the LBD facilitates high-affinity binding to estrogen ligands [13]. Ligand binding promotes ER dimerization and translocation to the nucleus. Efficient ER–DNA interaction is supported by pioneer factors (e.g., FOXA1, which has been confirmed in steroid-hormone-associated cancers, including ovarian cancer [14,15]), which promote chromatin accessibility at regulatory regions. Ligand binding induces conformational rearrangements in the ER, enabling recruitment of transcriptional coactivators and subsequent activation of target gene expression through enhanced chromatin accessibility, histone acetylation, and activation of RNA polymerase II transcriptional complexes [16,17]. In contrast, tethered ER signaling involves indirect DNA association via protein–protein interactions with other transcription factors, including AP-1 Fos/Jun complexes. Non-genomic estrogen signaling is mediated by membrane-associated ERs or G protein-coupled estrogen receptors (GPER), leading to rapid activation of intracellular signaling cascades such as MAPK and AKT pathways. Additionally, ligand-independent ER activation can occur through phosphorylation by various protein kinases, including PKA, PKC, and MAPK, further expanding the regulatory scope of estrogen signaling [1,16,17].

Although human exposure to BPA and ZEA is increasing as a consequence of widespread plastic pollution and climate change, which promotes the growth and mycotoxin production of filamentous fungi, the current scientific literature provides limited insight into the molecular effects of these compounds, particularly in the context of genomic events. Furthermore, most available studies have been conducted using animal models, whereas evidence from human studies remains comparatively scarce [2,10]. In the present study, we aimed to elucidate the genomic mechanisms underlying ZEA- and BPA-induced estrogenic responses. Our previous studies demonstrated that treatment of ERα-positive ovarian cancer cell lines with ZEA or BPA significantly enhanced cellular proliferation and migration, which was further confirmed by transcriptomic analyses [18,19]. Here, we comparatively characterized the ERα-binding landscapes in human ovarian cells exposed to ZEA, BPA, and physiological estradiol (E2) using chromatin immunoprecipitation followed by sequencing (ChIP-seq). ChIP-seq analysis is a powerful technique for the genome-wide identification of transcription factor binding sites and other chromatin-associated proteins [20]. This approach enabled the identification of ERα-bound genomic regions in response to ZEA and BPA exposure, which were subsequently integrated with previously generated RNA-seq transcriptomic data. Through this integrative analysis, we defined a core set of estrogen-responsive genes that are regulated by ERα and might contribute to the estrogenic response of human ovarian cells.

## 2. Materials and Methods

### 2.1. Culturing Conditions

The PEO1 human epithelial ovarian cell line was used in this study and was purchased from Merck (Darmstadt, Germany; ECACC, Salisbury, UK). The expression of ERα and estrogen sensitivity was confirmed in our previous studies [18]. Cells were cultured in RPMI1640 supplemented with 10% fetal bovine serum (FBS), 1% L-glutamine, 100 µg/mL streptomycin, and 100 U/mL penicillin (Corning, New York, NY, USA) at 37 °C, 90% humidity, and 5% CO_2_. In the genomic and transcriptional studies, cells were harvested by trypsinization and plated in the above-mentioned culture medium. After 24 h, the medium was replaced with PRF-RPMI1640 supplemented with 5% DCC-FBS and further incubated for 24 h. This step was necessary in order to decrease the confounding effect of phenol red or estrogens present in the conventional medium. Note that in order to make the ERα extremely sensitive to estrogen treatment, this incubation period was increased to 72 h in the ChIP-seq analysis. After the incubation period, cells were treated with E2 (10 nM), ZEA (10 nM), and BPA (100 nM) as in our previous experiments [19]. This time was designated as 0 h. In selected experiments, cells were pre-incubated with 1 µM methyl-piperidinopyrazole (MPP) for 2 h prior to treatment.

### 2.2. Chromatin Immunoprecipitation Followed by Sequencing (ChIP-Seq)

In the ChIP-seq analysis, the samples were generated as follows: 10^6^ cells were plated into T-75 flasks; treated with E2, ZEA, and BPA; and further incubated for 1 h as described above. In the ChIP-seq experiment, vehicle-treated cells served as controls, and all experimental conditions were analyzed in biological duplicates. The ChIP experiment was performed as described previously [21] using an anti-ERα antibody (Sigma, St. Louis, MO, USA, cat. no. 06-935). Precipitated DNA was isolated using the NucleoSpin Gel and PCR Clean-up Kit (Macherey-Nagel, Düren, Germany) according to the manufacturer’s instructions. The concentration of ChIP-DNA was determined using a Qubit fluorometer (Thermo Fisher Scientific, Waltham, MA, USA). For the sequencing analysis, cDNA libraries were prepared with Ovation Ultralow Library Systems V2 (Tecan, Männedorf, Switzerland) according to the manufacturer’s instructions. Fragment size distribution of the libraries was determined with a BioAnalyzer 2100 (Agilent, Santa Clara, CA, USA). Sequencing was performed on the Illumina NextSeq 500 instrument (Illumina, San Diego, CA, USA) with single-read 75 bp sequencing reads. Raw reads were aligned to the human reference genome GRCh38.p13. Sequencing yielded 12–16 million reads per sample, with >96% of reads mapped to the reference genome. Sequencing and library quality metrics (Q30, duplication rate, FriP score, etc.) are provided in Supplementary Materials Table S1.

### 2.3. Analysis of ChIP-Seq Data

The primary analysis of ChIP-seq reads was carried out using our ChIP-seq analysis pipeline [22]. MACS2 was used for predicting ‘narrow peaks’ with the following specific parameters: q-value cut-off (q) = 0.001 and subpeaks deconvolved within each peak (--call-summits). Artifacts were removed using the ENCODE blacklist (https://www.nature.com/articles/nature11247; accessed on 8 June 2026). Regions with accession prefixes of NW and NT were excluded; only regions starting with NC (complete genomic assembly) were used for the analyses. Sample quality metrics are provided in Supplementary Materials Table S1.

Consensus peak sets were determined for each condition containing the common peaks of the two replicates. Normalized tag counts (expressed as reads per kilobase per million mapped reads, RPKM) were calculated using bamtools, bedtools, and awk. Integrative Genomics Viewer (IGV, Broad Institute) was used for data browsing and creating representative snapshots [23]. Genome coverage files (BedGraphs) were converted into tdf files using igvtools with the ‘toTDF’ option. Read distribution (RD) histograms were generated by HOMER (Hypergeometric Optimization of Motif EnRichment) [24] using tag directories and BED files and then visualized by Java TreeView. Positions of transcription start sites (TSS) were obtained from the UCSC database. De novo motif discovery was performed using the HOMER tool.

ERα ChIP-seq profiles were integrated with our previously generated RNA-seq data [19]. Functional annotation of the identified gene sets was performed using the Network Analyst platform (https://www.networkanalyst.ca/; accessed on 7 September 2026), based on the STRING Interactome and Reactome databases [25].

### 2.4. mRNA Isolation and Quantification by qPCR

To analyze transcriptomic responses in PEO1 cells, 10^5^ cells were plated into 24-well plates and treated at time 0 h with E2, ZEA, or BPA, according to our previous experiments [18,19]. Total RNA was extracted at 0, 2, 6, and 24 h following treatment in time-course gene induction experiments or at 4 h post-treatment in experiments involving MPP pre-treatment using the Quick-RNA MiniPrep Kit (Zymo Research, Irvine, CA, USA) according to the manufacturer’s instructions. Reverse transcription was performed using 500 ng of total RNA as the input with the Maxima First Strand cDNA Synthesis Kit (Thermo Fisher Scientific), following the manufacturer’s instructions. RNA and cDNA concentrations were quantified using a NanoDrop LITE Spectrophotometer (Thermo Fisher Scientific, Waltham, MA, USA). *GREB1*, *RASGRP1*, *CISH*, *RBBP8*, *KCNF1*, *LONRF2*, *ACTRT3* and *GAPDH* expression levels were determined by quantitative PCR (qPCR) using Maxima™ SYBR Green qPCR Master Mix (Thermo Fisher Scientific) in a LightCycler 96 system (Roche, Basel, Switzerland), according to the manufacturer’s recommendations. Primer sequences are listed in Table 1. Gene expression values were normalized to *GAPDH* expression, and data from four independent experiments were included in the analysis, consistent with our previous studies [18,19]. Relative expression levels were calculated using the 2^−ΔΔCt^ method, where ΔΔCt = ΔCt (treated sample) − ΔCt (control sample). Statistical analyses and graphical representations were generated using GraphPad Prism 7.0.

## 3. Results

### 3.1. Identification of ERα-Binding Regions in Cells Activated by E2, ZEA, and BPA Using ChIP-Seq

Although estrogen signaling can be mediated through multiple genomic and non-genomic pathways, ERα-dependent transcriptional regulation is considered the predominant mechanism in estrogen-responsive cells [16]. In addition to the endogenous estrogen E2, both ZEA and BPA are able to exert estrogenic activity through ERα binding [16]. To characterize ERα-binding patterns in cells activated by E2, ZEA, or BPA, ChIP-seq analysis was performed. Cells were treated with vehicle or ligands for 1 h. Following stimulation, cells were crosslinked, sonicated, and processed for ChIP-seq. Chromatin fragments were immunoprecipitated using an ERα-specific antibody under experimental conditions identical to those applied in our previous studies [18,19,26].

E2, ZEA, and BPA exposure induced extensive genome-wide ERα occupancy across ovarian cells (Figure 1). Genome-wide peak calling identified a greater number of peaks in the E2- and ZEA-treated samples than in the BPA-treated samples (Figure 2). The median peak width was 416 bp and 431 bp in the E2-treated, 423 bp and 419 bp in the ZEA-treated, and 388 bp and 281 bp in the BPA-treated replicates (Supplementary Materials Table S1). Consensus peak sets for each ligand were defined as peaks detected in both corresponding biological replicates. This analysis identified 63, 2214, 3200, and 656 reproducible ERα-binding regions in cells treated with vehicle (control), E2, ZEA, and BPA, respectively (Figure 2A, Supplementary Materials Table S2). We performed *de novo* motif enrichment analysis of ERα-bound regions. The canonical ERE motif was consistently identified as the top enriched motif across all treatment conditions except for the control (Figure 2B).

### 3.2. E2 and ZEA Elicit More Robust ERα-Mediated Responses than BPA

Next, we compared ERα-binding profiles across the different treatment conditions. Comparative peak analysis revealed a substantial degree of overlap between treatments. We identified 641 ERα-bound regions that were consistently detected in all three treatment conditions (Figure 3A, Supplementary Materials Table S3). Notably, 98% of the peaks responsive to BPA exposure also exhibited induction following E2 and/or ZEA treatment, indicating that only 7 peaks were uniquely associated with BPA exposure. In addition to a core peak set detected across all three treatment conditions (*n* = 641), three other large peak sets were identified: peaks detected in both E2- and ZEA-treated cells (*n* = 1450) and peaks detected uniquely in E2-treated (*n* = 123) or ZEA-treated cells (*n* = 1105) (Figure 3A; Supplementary Materials Table S3). Although the common set of ERα-binding regions was identified in all ligand-treated conditions, differences in ERα-binding signal intensity were detected at these regions (Figure 3B).

To determine quantitative differences in ERα occupancy in all ERα-binding peak sets, we compared ERα signals (RPKM values) detected in the four conditions (control, E2-, ZEA-, and BPA-treated). This led to two key observations. First, at peaks detected across all three conditions, signal intensity was higher in E2- or ZEA-treated cells than in BPA-treated cells, suggesting stronger binding at shared loci (Figure 3C, Supplementary Materials Table S3). Second, ERα signal intensity in a given treatment was generally higher within the common peak set than in the E2-only, ZEA-only, or E2–ZEA-specific peak sets (Figure 3C–F, Supplementary Materials Table S3). These results suggest stronger and more stable ERα binding at these shared regulatory regions. It should be noted that low-intensity ERα peaks were also detected in vehicle-treated samples. This observation may be explained by previous reports demonstrating that ERα can be pre-recruited to specific genomic loci prior to ligand stimulation [27,28].

### 3.3. Correlation Between ChIP-Seq Data and Transcriptomic Profiling

Our previous RNA-seq analyses, performed under the same experimental conditions, characterized the transcriptomic programs induced by E2, ZEA, and BPA [19]. To investigate the relationship between transcriptional responses and ERα occupancy, we integrated this dataset with the corresponding ERα ChIP-seq profiles. Comparative analysis of ChIP-seq and RNA-seq datasets revealed a strong relationship between the patterns of ERα binding and gene expression changes (Figure 4). First, we collected the previously identified 365 genes that were significantly upregulated (log_2_FC ≥ 1) by any of the ligands [19]. Next, ERα-binding regions located within the transcription start site (TSS) ± 50 kb window of these upregulated genes were identified, resulting in 162 ERα-binding regions associated with 109 upregulated genes (Figure 4A,B, Supplementary Materials Table S4). Among these ERα-binding regions, 17 were associated with two genes (Supplementary Materials Table S4). These results suggest that ERα is involved in the induction of these 109 genes by E2, ZEA, and/or BPA.

Based on RPKM values, the signal intensity of these 109 genes was highest in the E2-treated samples, was comparable to that observed in the ZEA-treated samples, and was lower after BPA exposure (Figure 4C,D). Fold-change (FC) values were calculated based on ERα-binding signals and gene induction levels in the treated samples compared to the untreated controls. This also suggested higher gene induction in response to E2 and ZEA than to BPA (Figure 4E,F). Furthermore, genes commonly induced by all three treatments exhibited significantly greater fold changes in response to E2 or ZEA than genes induced only by E2 and ZEA or exclusively by either E2 or ZEA, according to the RNA-seq analysis (Figure 4G,H). Collectively, these findings demonstrate a relationship between the pattern of ERα occupancy and gene regulation, supporting the hypothesis that ERα-binding patterns are not random but are associated with corresponding changes in mRNA expression.

Furthermore, integration of the RNA-seq and ChIP-seq datasets identified 28 genes that were induced by E2, ZEA, and BPA and whose ERα-dependent regulation was also supported by ChIP-seq (Table 2, Supplementary Materials Table S4). In the TSS ± 50 kb regions of *AGT*, *DHRS2*, *GREB1*, *KCNF1*, *LONRF2*, *PDGFRL*, *PIPOX*, *RASGRP1*, *RBBP8*, and *SLC7A2*, more than one ERα peak was identified (Table 2).

### 3.4. The Stronger Transcriptional Response Induced by E2 and ZEA than by BPA Remained Stable over Time and Was Suppressed by the Application of an ERα Antagonist

Among the 28 previously identified genes, six (*GREB1*, *RASGRP1*, *CISH*, *RBBP8*, *KCNF1*, and *LONRF2*) were selected for further analysis (Figure 5). Their induction following treatment with E2, ZEA, and BPA was assessed by RT-qPCR. To capture both early and late transcriptional responses, gene expression was analyzed at 2, 6, and 24 h after treatment. Estrogen exposure induced rapid upregulation of all six genes in response to each compound tested, as expected (Figure 6). Consistent with our previous observations, E2 and ZEA elicited significantly stronger gene induction than BPA. Notably, the magnitude of this difference remained unchanged at later time points, indicating that the reduced inductive capacity of BPA was sustained over time (Figure 6).

We also included *ACTRT3* in our analysis as a negative control gene, as it is not known to be involved in ERα-mediated estrogen signaling. Consistent with this assumption, *ACTRT3* did not exhibit induction following E2, ZEA, or BPA treatment (Supplementary Materials Figure S1). To further support the ERα dependence of *GREB1*, *RASGRP1*, *CISH*, *RBBP8*, *KCNF1*, and *LONRF2* induction by E2, ZEA, and BPA, cells were pre-treated with the selective ERα antagonist MPP [29] before ligand exposure. Treatment with 1 µM MPP significantly reduced gene induction, indicating that ERα contributes to the observed transcriptional response (Figure 7).

### 3.5. Estrogen-Responsive Genes Are Highly Interconnected in the Regulation of Cell Cycle

To obtain a comprehensive understanding of the biological functions and molecular interactions associated with the identified genes, we performed an integrated functional annotation analysis incorporating both protein–protein interaction (PPI) network analysis and gene set enrichment approaches. Initially, all 109 identified genes were included in the analysis. This was conducted using the Network Analyst platform, based on the STRING Interactome database with experimentally validated interactions and a high-confidence score threshold (900). The resulting PPI network revealed extensive interactions, comprising 395 nodes, 456 edges, and 23 seeds (Figure 8). We identified *NOL6*, *FOS*, and *NOD2* as central hub genes that exhibited the highest degree and betweenness centrality values, indicating their potential regulatory importance within the network (Table 3).

To further characterize the biological relevance of these genes, functional enrichment analyses were performed using the Reactome database. Reactome analysis demonstrated a significant enrichment of pathways involved in cell-cycle regulation, highlighting the potential contribution of the identified genes to cell proliferation and cell-cycle control mechanisms (Figure 9A). Finally, gene set enrichment analysis was performed using the core set of 28 genes. This analysis identified pathways related to amino acid transport as the most significantly enriched biological processes. In addition, estrogen-responsive gene expression programs emerged among the top enriched pathways (Figure 9B).

## 4. Discussion

Despite the increasing human exposure to xenoestrogens, their molecular mechanisms of action at the genomic level remain poorly understood. In this study, we compared the ERα-mediated effects of E2, ZEA, and BPA in human ovarian cancer cell cultures using ChIP-seq analysis. Our results demonstrated that all three compounds induced ERα binding at multiple genomic loci across the human genome. To the best of our knowledge, this represents the first genome-wide characterization of ERα binding in response to ZEA treatment, as well as the first study investigating BPA-induced ERα binding in human ovarian cell cultures.

Our results demonstrate that the transcriptomic response induced by ZEA was comparable to that elicited by physiological E2. Consistent with this observation, our previous studies demonstrated that ZEA-induced cell proliferation and migration were comparable to those elicited by E2 [18,19]. Our data further support the role of ERα in the transcriptional response elicited by ZEA, which is in good agreement with previous reports showing that ZEA directly interacts with ERα and exhibits potent agonistic activity in ERE-luciferase reporter assays across multiple cell lines [30]. Furthermore, treatment with 10 nM ZEA induced a comparable upregulation of estrogen-responsive genes in MCF-7 cells [31], and ZEA proved to be able to induce epigenetic modifications in several studies [32]. Consistent with these findings, ChIP-seq and RT-qPCR analyses in the present study confirmed an ERα-mediated transcriptional induction profile similar to that of E2 that remained stable over time. This finding highlights the importance of this mycoestrogen, as ZEA contamination of food commodities is considered widespread and frequently occurs at relatively high levels [33]. The potential health risks associated with ZEA exposure are further supported by human biomonitoring studies demonstrating the presence of ZEA in various body fluids. Although the ZEA concentrations reported in human serum and follicular fluid are generally lower than those applied in the present study, substantially higher levels have been detected in urine samples, in endometrial cancer tissues, or in blood samples of patients with colorectal cancer [34,35,36,37].

In contrast to the pronounced estrogenic activity of ZEA, BPA elicited substantially weaker ERα-mediated estrogenic effects. This observation is consistent with previous reports demonstrating that BPA exhibits a markedly lower potency in inducing ERα-mediated gene transcription, thereby classifying it as a weak estrogen [38,39]. Several mechanisms have been reported previously to account for its reduced estrogenic potency: (i) BPA displays a significantly lower binding affinity for both ERα and ERβ than E2 [40]; (ii) BPA interacts differently with the ligand-binding domain of ERs, resulting in less efficient receptor activation compared with E2 [41,42], and consequently, substantially higher BPA concentrations are required to elicit an ERE-mediated transcriptional response [30]; and (iii) the BPA–ERα complex exhibits altered transcriptional coactivator recruitment and nucleosome positioning, thereby limiting downstream transcriptional activation [43,44,45]. Our data further indicate that the ERα-mediated response induced by BPA was reduced both in magnitude and in ERα occupancy at ERα-binding genomic regions, and the lower transcriptional inductive capacity of BPA remained consistent over time. The observation that BPA-induced transcriptional activation was concentrated at loci displaying strong ERα occupancy, together with the pronounced induction of the corresponding genes, suggests that BPA may preferentially increase the expression of highly estrogen-responsive genes. Our findings are in good agreement with previous reports demonstrating a weaker genome-wide ERα-mediated response to BPA in T-47D and ECC-1 cells [46]. Importantly, ERα-binding profiles displayed extensive cell-type specificity, suggesting that estrogen signaling is mediated through distinct context-dependent transcriptional programs [17,46]. Consequently, integrative studies encompassing diverse cellular models will be essential for accurately assessing the impact of xenoestrogens on human health. It is also important to note that although BPA exerts relatively modest estrogenic effects at low physiological exposure levels, its biological relevance may become more pronounced under conditions characterized by reduced endogenous E2 concentrations, such as during infancy and childhood [5].

Integration of our ChIP-seq and previous RNA-seq datasets enabled the identification of 109 estrogen-responsive genes that might be regulated by ERα in human ovarian cells. Among these, we identified the core gene set, including 28 highly estrogen-responsive genes, six of which were further validated by RT-qPCR. The induction of *GREB1*, *CA12*, *NPY1R*, *OLFML3*, *RBBP8*, *SLC7A2*, and *SLC7A5* by BPA has also been reported previously in breast cancer cell lines [45,47,48,49,50,51]. However, only the induction of *NPY1R* was previously reported in response to ZEA exposure [31]. Furthermore, the activity of *GREB1*, *NPY1R*, *PLAT*, *SLC19A2*, *SLC7A2*, *SLC7A5*, *RBBP8*, and *PDGFRL* correlated with ER positivity and/or endocrine resistance in breast cancer [52,53,54,55,56,57,58].

Functional annotation of the identified gene sets revealed significant enrichment of biological processes associated with cell-cycle regulation, amino acid transport, and ribosome biogenesis. These findings are consistent with previous observations demonstrating that both estrogens and xenoestrogens promote cellular proliferation in several cell lines [18,59,60,61,62]. The increased biosynthetic potential might be supported by the upregulation of ribosome biogenesis and amino acid transport pathways, which together facilitate elevated protein synthesis capacity [63,64]. Collectively, these findings support the hypothesis that estrogenic and xenoestrogenic exposures may contribute to cancer development [2,9].

## 5. Conclusions

Despite the well-recognized threat posed by endocrine-disrupting compounds to human health, their molecular mechanisms of action remain poorly understood from a genomic perspective. This knowledge gap is particularly pronounced for ZEA. Our findings suggest that ZEA induces ERα binding to genomic regions with an efficiency comparable to that of the physiological ligand E2. In contrast, BPA is less potent in activating the ERα-dependent transcriptional program, with only the most highly estrogen-responsive loci exhibiting detectable transcriptional responses to this xenoestrogen. To the best of our knowledge, this is the first study to characterize the genomic actions of BPA in human ovarian cells and of ZEA in any experimental model system. Moreover, we identified 28 highly estrogen-responsive genes that were consistently induced by both ZEA and BPA. We emphasize that ERα-mediated estrogen signaling is considered to be highly cell-type-dependent. An important limitation of this study is that the analyses were restricted to a single ovarian cell line; therefore, the generalizability of these findings to other ovarian cell types requires further validation to delineate the context-specific mechanisms underlying ERα-mediated responses. Furthermore, systematic investigation of ERα cofactor interactions, ERα occupancy dynamics, and chromatin accessibility changes would provide important mechanistic insights and represent a promising direction for future research.

## Figures and Tables

**Figure 1:**
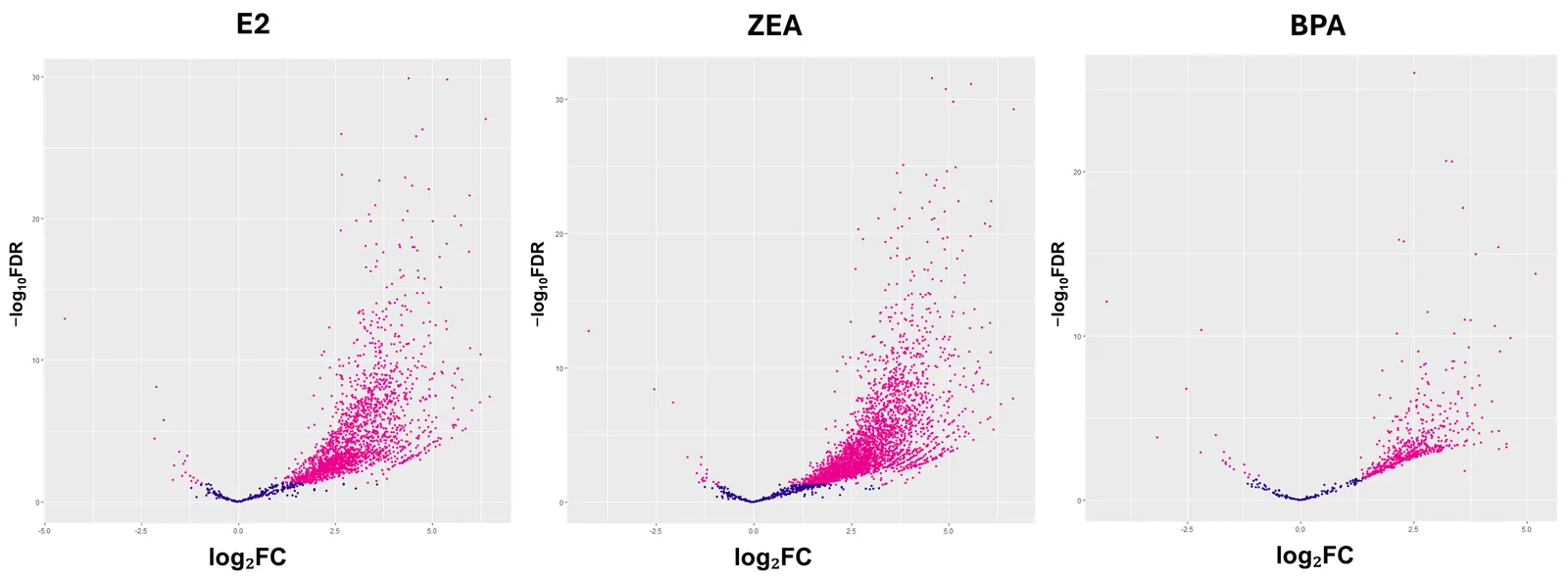
Volcano plots showing differential ERα occupancy in E2-, ZEA-, and BPA-treated ovarian cells relative to control cells. Volcano plots were generated using DiffBind with DESeq2, and regions with FDR < 0.05 (pink nodes) were considered to show significant differential occupancy.

**Figure 2:**
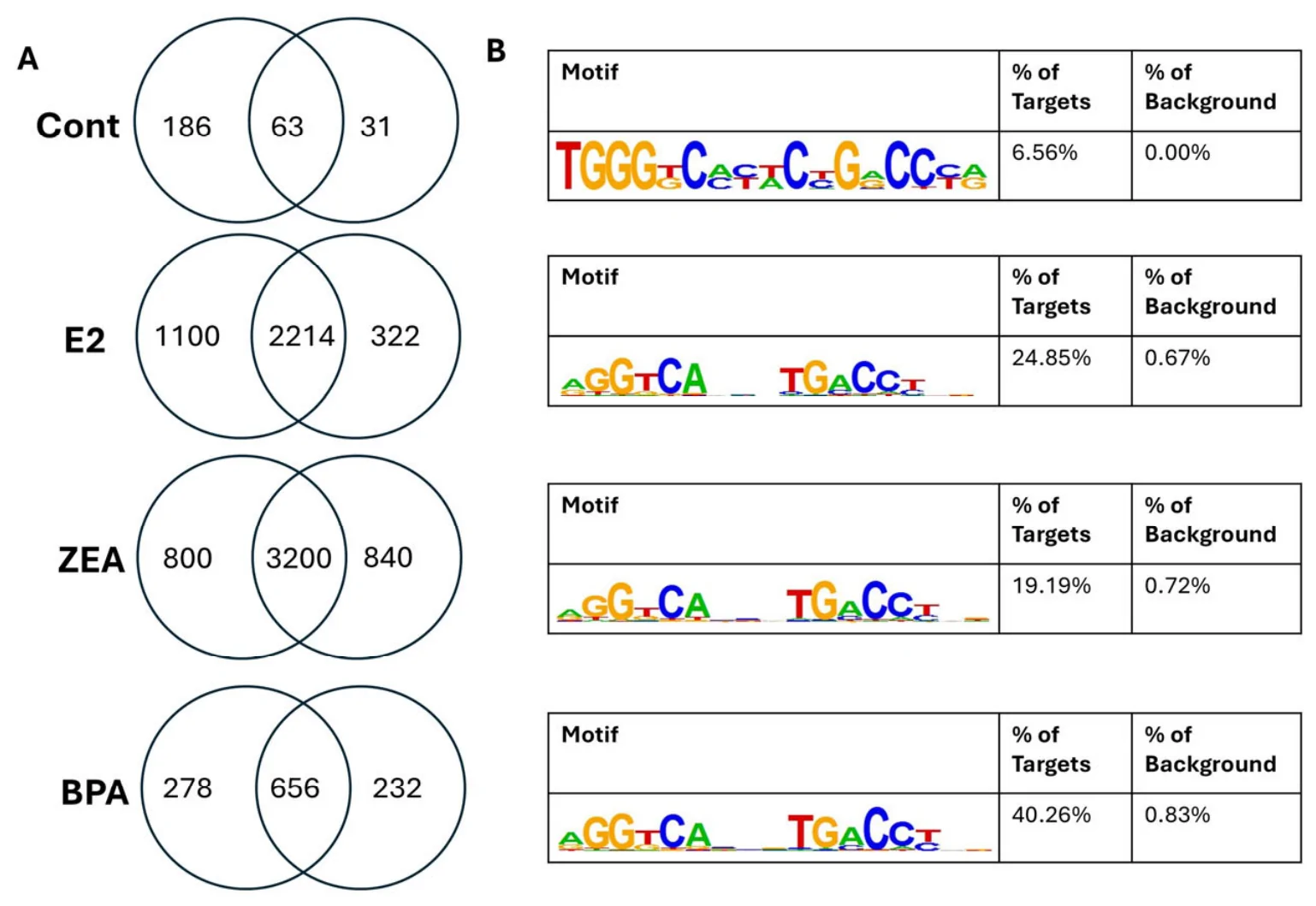
Identification of ERα-binding regions in PEO1 cells. (**A**) Overlap of ERα-binding regions in two replicates of PEO1 cells stimulated with vehicle (Cont), E2, ZEA, and BPA, as determined by ChIP-seq. (**B**) Results of *de novo* motif discovery under ERα peaks from ChIP-seq data using HOMER. “% of Targets” refers to the ratio of the peaks containing the motif, and “% of Background” refers to the ratio of a random background. The top motif is presented for each condition.

**Figure 3:**
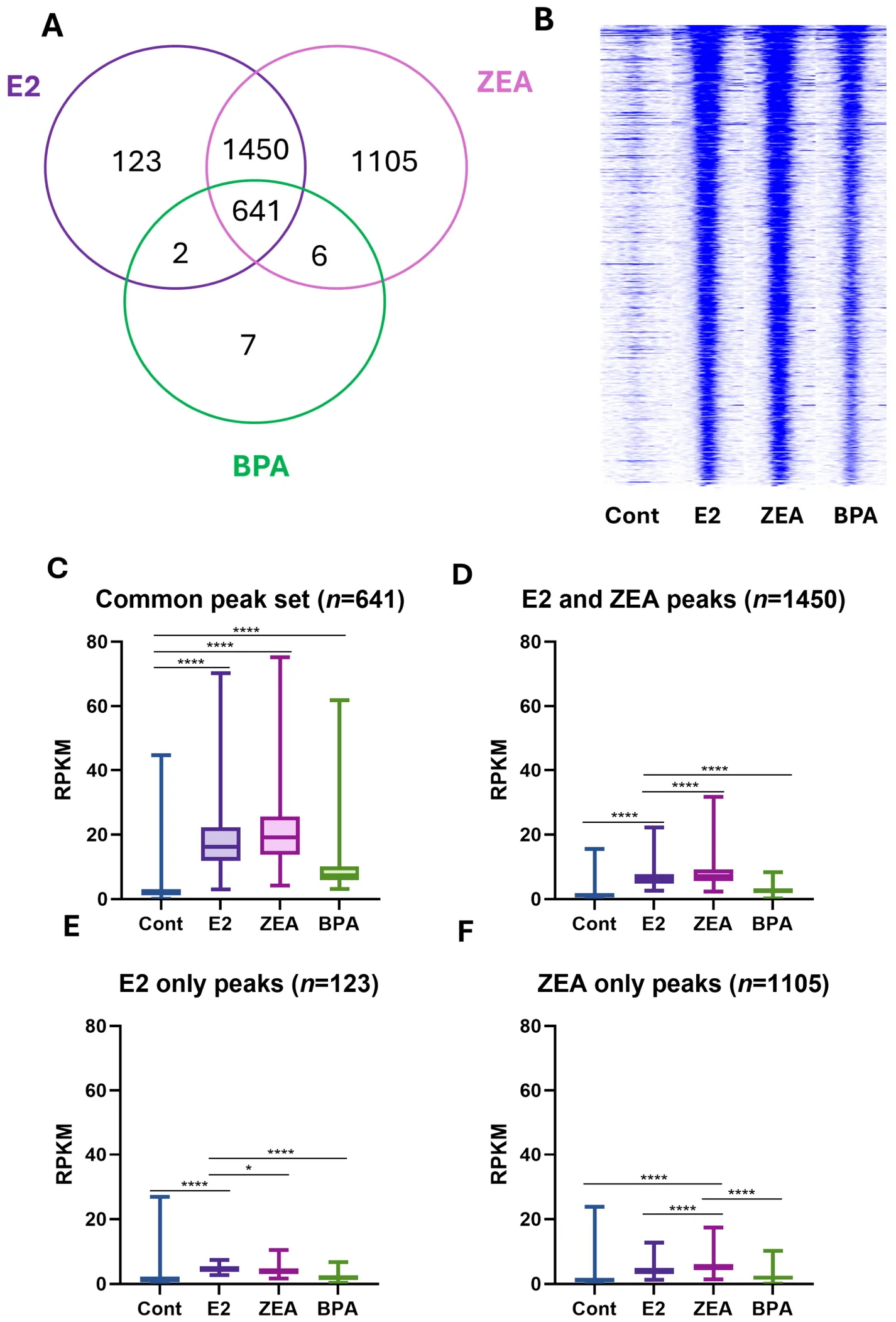
Comparison of ERα-binding patterns in PEO1 cells stimulated with ERα agonists. (**A**) Overlap of ERα-binding regions in PEO1 cells stimulated with E2, ZEA, and BPA, as determined by ChIP-seq. (**B**) Read distribution (RD) plot of ERα signals in the common peak set (*n* = 641) across 2 kb windows in the four conditions (control, E2-, ZEA-, and BPA-treated). (**C**–**F**) ERα-occupancy signals identified by ChIP-seq, according to the RPKM values in the four peak sets (common peak set, E2 and ZEA, E2 only, and ZEA only) in the four conditions (control, E2-, ZEA-, and BPA-treated). * *p* ≤ 0.05, **** *p* ≤ 0.0001 (one-way ANOVA followed by Dunnett’s multiple comparisons test).

**Figure 4:**
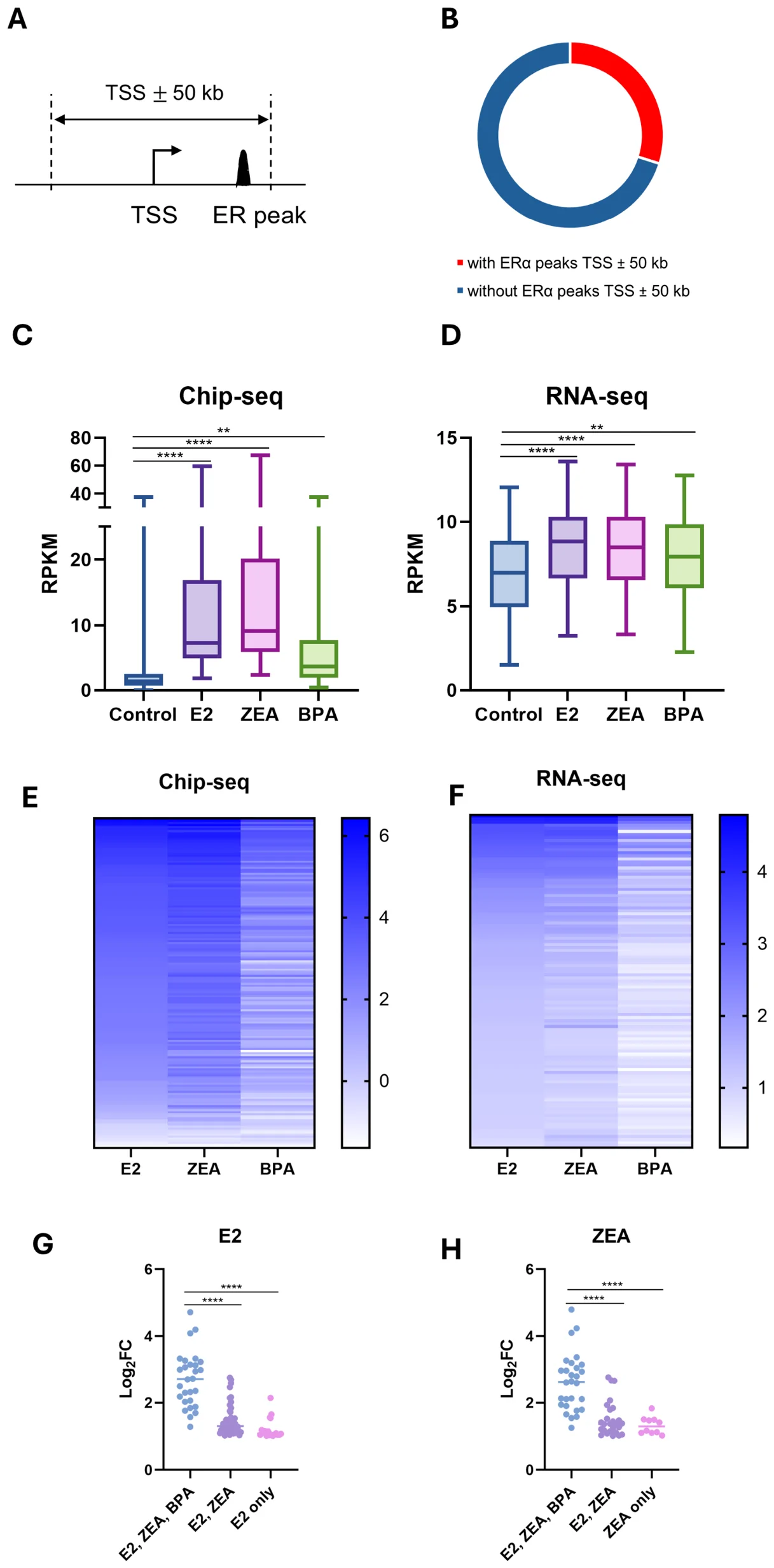
Correlation between RNA-seq and ChIP-seq data. (**A**) ERα-binding regions located within the transcription start site (TSS) ± 50 kb window of upregulated genes according to RNA-seq were identified. (**B**) Proportion of genes induced by any of the ER ligands with or without identified ERα peaks. (**C**) ERα-occupancy signal intensity (RPKM values) according to ChIP-seq for the 162 identified peaks. ** *p* ≤ 0.01, **** *p* ≤ 0.0001 (one-way ANOVA followed by Dunnett’s multiple comparisons test). (**D**) Signal intensity (RPKM values) according to RNA-seq, for the 109 identified genes. ** *p* ≤ 0.01, **** *p* ≤ 0.0001 (one-way ANOVA followed by Dunnett’s multiple comparisons test). (**E**) Heatmap of ERα signal fold change in response to E2, ZEA, and BPA in the case of the 162 identified peaks. Log_2_FC values are presented. (**F**) Heatmap of gene induction in response to E2, ZEA, and BPA in the case of the 109 identified genes. Log_2_FC values are presented. (**G**) Comparison of the log_2_FC values of genes induced in response to E2, ZEA, and BPA; in response to E2 and ZEA; or in response to E2 only. The log_2_FC values of E2-treated samples are shown. (**H**) Comparison of the log_2_FC values of genes induced in response to E2, ZEA, and BPA; in response to E2 and ZEA; or in response to ZEA only. The log_2_FC values of ZEA-treated samples are shown.

**Figure 5:**
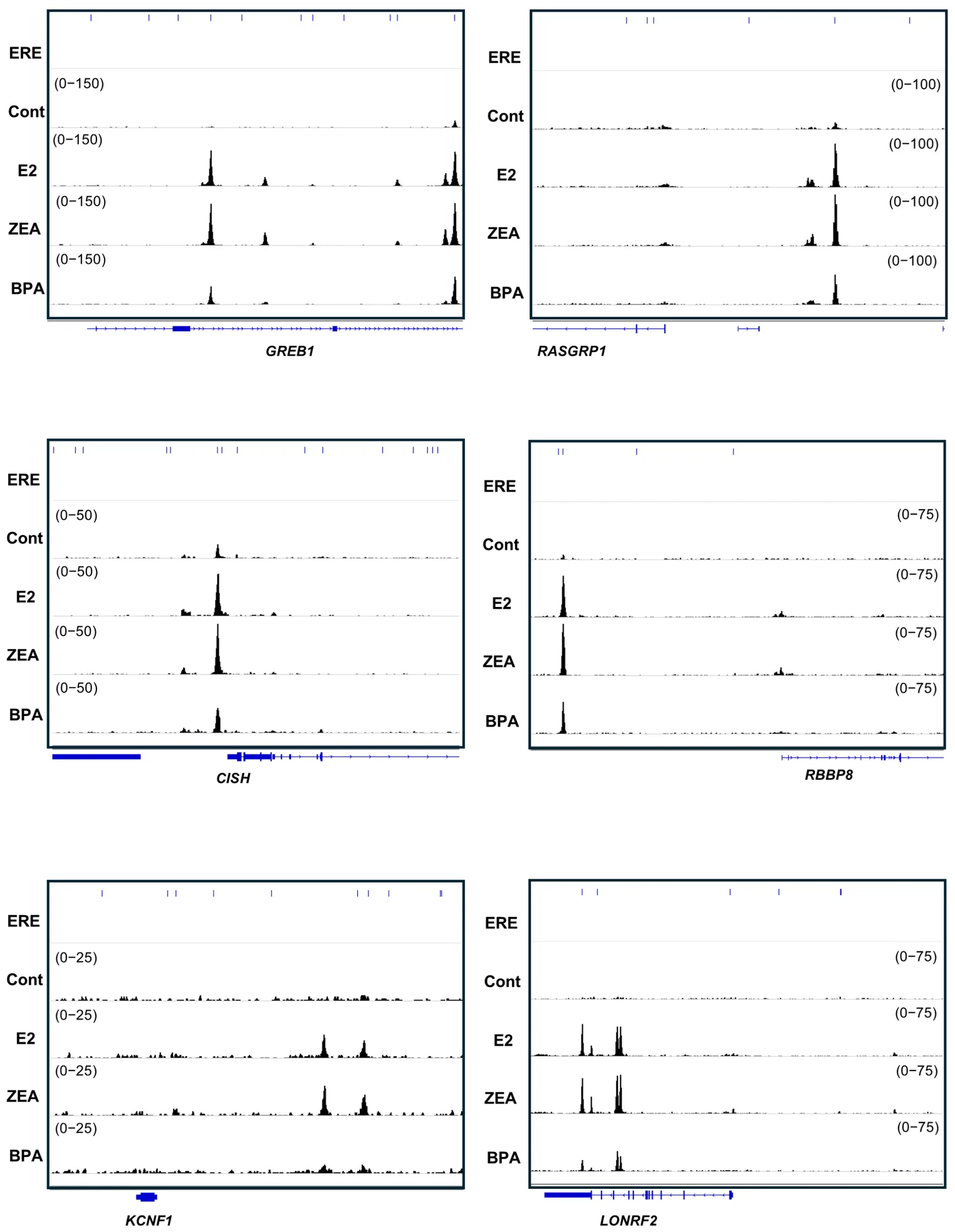
Genome browser view of ERα binding in the *GREB1*, *RASGRP1*, *CISH*, *RBBP8*, *KCNF1*, and *LONRF2* genomic regions. ERα binding was determined in the four conditions (control, E2-, ZEA-, and BPA-treated). Identified ERE sequences are also indicated.

**Figure 6:**
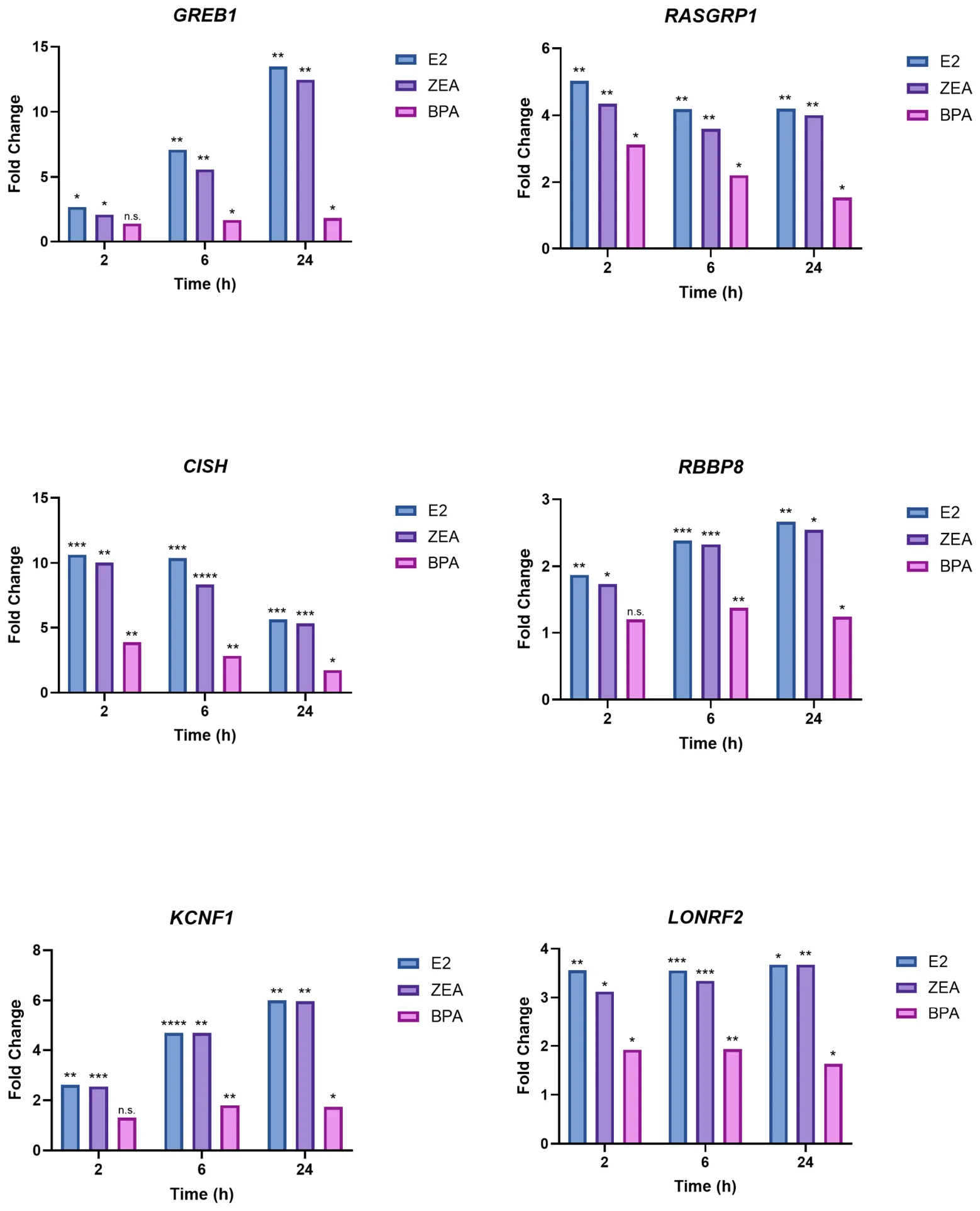
Analysis of time-dependent gene induction in response to E2, ZEA, and BPA exposure by RT-qPCR. Gene expression changes are presented as fold changes relative to the vehicle-treated control and were calculated from four independent experiments. Statistical significance was assessed using ΔCt values. n.s.: non-significant; * *p* ≤ 0.05; ** *p* ≤ 0.01; *** *p* ≤ 0.001; **** *p* ≤ 0.0001 (two-way ANOVA followed by Dunnett’s multiple comparisons test).

**Figure 7:**
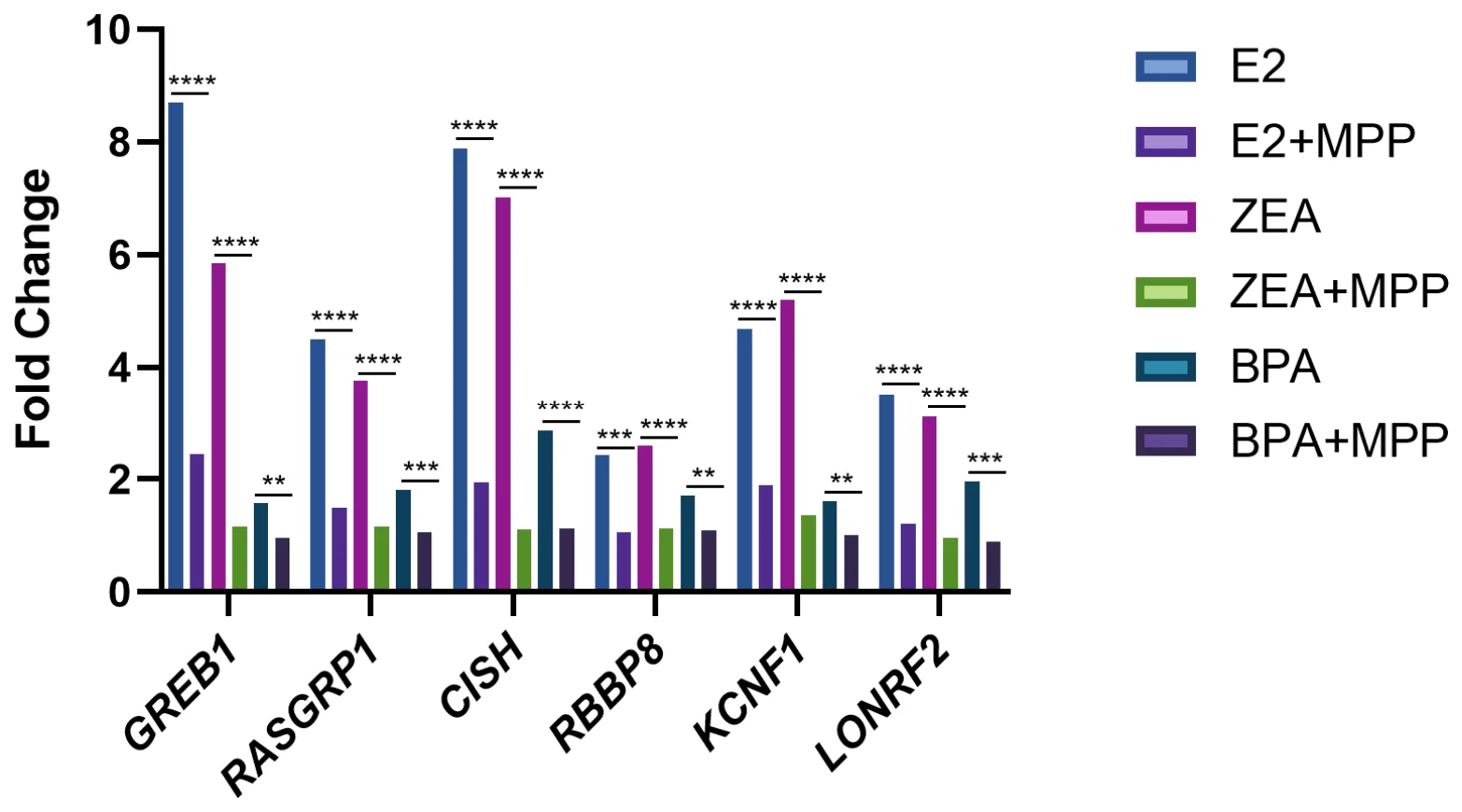
Analysis of the effects of MPP on ERα-mediated gene induction in response to E2, ZEA, or BPA. Gene expression levels are presented as fold changes relative to vehicle-treated controls for E2-, ZEA-, or BPA-treated samples and relative to MPP-treated cells for samples co-treated with MPP and E2, ZEA, or BPA. RNA was isolated 4 h after treatment. Statistical significance was assessed using ΔCt values from four independent experiments. ** *p* ≤ 0.01; *** *p* ≤ 0.001; **** *p* ≤ 0.0001 (Student’s *t*-test).

**Figure 8:**
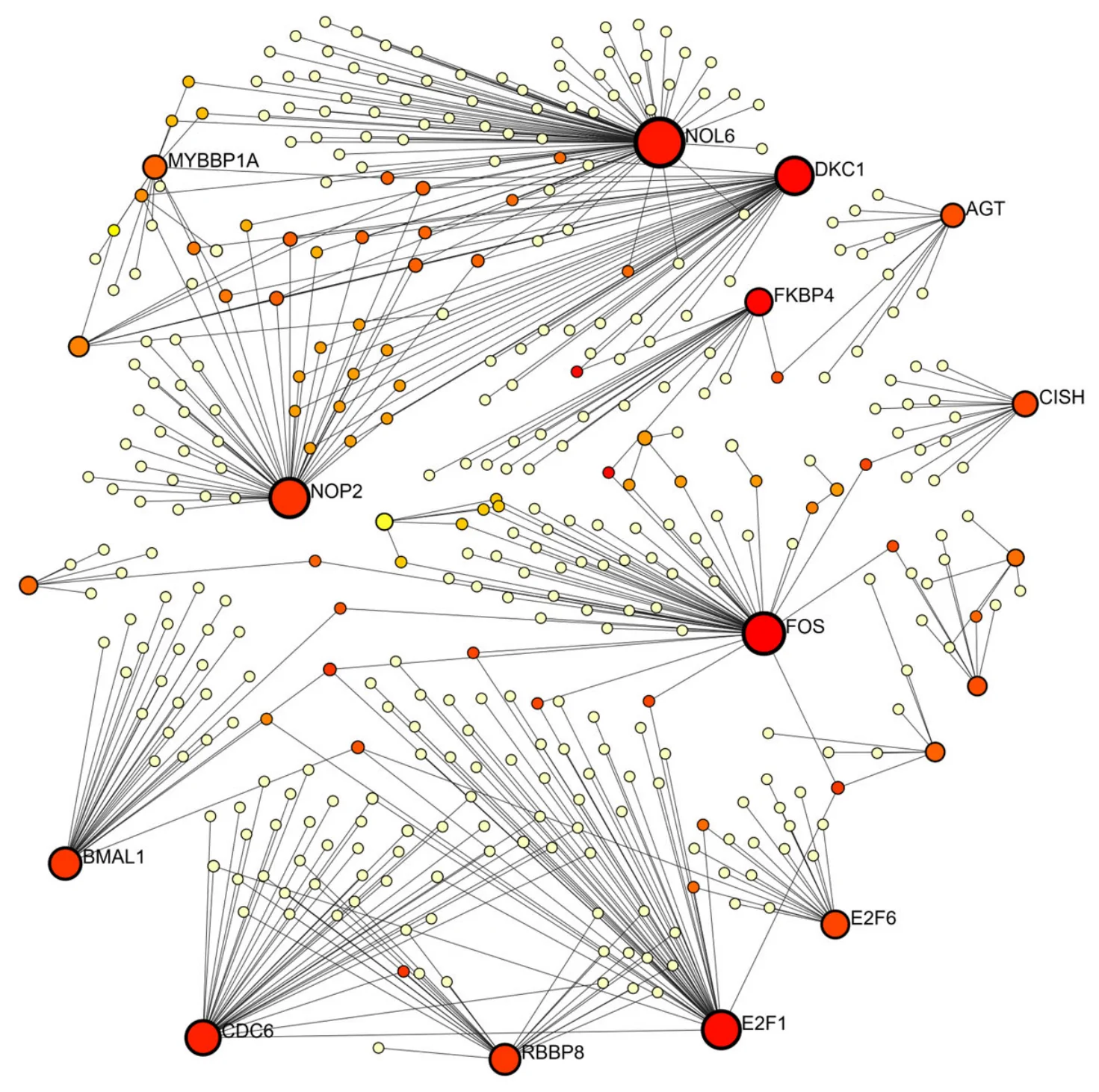
Network analysis of the 109 identified genes. The largest protein–protein interaction (PPI) network constructed between the protein products of the 109 identified genes is presented.

**Figure 9:**
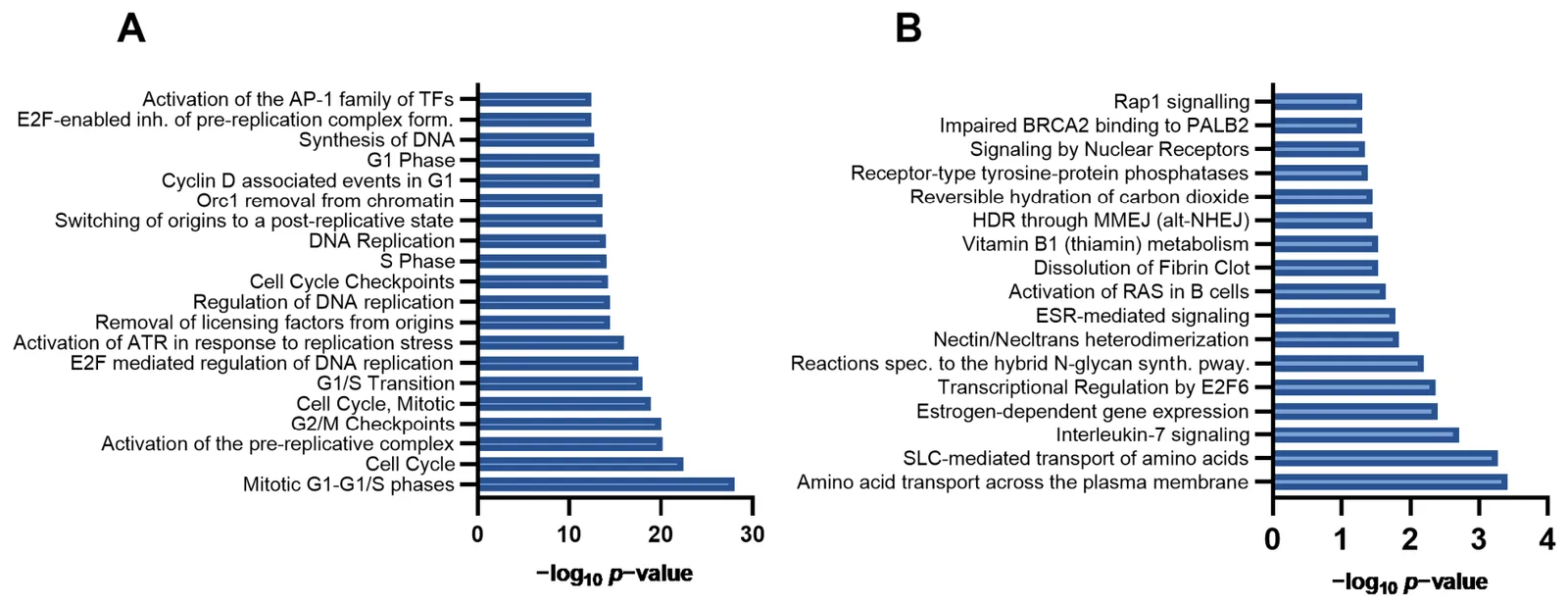
Functional enrichment analysis of the identified genes. (**A**) Functional annotation analysis of the interaction network comprising the 109 identified genes, based on the Reactome database. (**B**) Functional annotation analysis of the core gene set, including 28 identified genes, based on the Reactome database.

**Table 1:** Primer sequences used for the quantification of gene expression by RT-qPCR.

	Forward Primer	Reverse Primer
*GREB1*	TCTTGCACAATTCCATCGAG	GTCCACTCGGCTACCACCT
*RASGRP1*	CTGGGCAAGGCGAGAGAG	TTCGGAACTGGGTGATGTGG
*CISH*	TCCAGCCGAGTCCCCAC	GTACCCTCTGCCACCTCCTC
*RBBP8*	CACTCAGACTTGTATGGAAAGAGG	TCCTTCTGTTTCTGTTTCAACG
*KCNF1*	CTGCTCATCCTCGTCTCGTC	GAAGTGCAGCTTGTTGGGTG
*LONRF2*	TGGAGCTGGCTCCTGATGATAA	CCCAATCCAGAAAGAGCCTGA
*ACTRT3*	GACAACGGCTCGGGAATGAT	AATGAGACCACGCTCCACTG
*GAPDH*	CACCCACTCCTCCACCTTT	GCCAAATTCGTTGTCATACCA

**Table 2:** Core set of genes induced by E2, ZEA, and BPA according to RNA-seq analysis, with ERα peaks identified in the TSS ± 50 kb regions. Fold-change (FC) values were calculated relative to the vehicle-treated controls. Note that for some genes, more than one peak was identified. In these cases, all identified peaks are listed.

Gene Symbol	Peak	FC E2	FC ZEA	FC BPA
*AGT*	peak1	33.63	50.95	14.71
	peak2	6.36	9.23	4.14
*ARHGAP26*		15.54	18.50	8.20
*CA12*		4.51	3.22	0.33
*CADM1*		34.53	44.95	14.12
*CISH*		4.07	4.08	2.54
*DHRS2*	peak1	7.38	7.64	1.23
	peak2	86.24	72.39	30.33
*ERICH3*		11.34	14.44	5.29
*GREB1*	peak1	27.58	31.17	11.88
	peak2	10.43	15.97	4.33
	peak3	17.09	19.30	4.10
	peak4	62.15	45.04	9.84
	peak5	11.03	15.68	3.85
	peak6	5.63	6.32	3.99
*KCNF1*	peak1	30.30	40.22	11.47
	peak2	2.42	3.40	1.00
*LONRF2*	peak1	29.99	34.43	10.98
	peak2	6.73	9.65	2.19
	peak3	15.82	19.30	9.61
	peak4	24.60	29.36	9.97
	peak5	8.03	9.65	3.82
*MGAT3*		0.65	0.80	0.98
*NPY1R*		6.73	7.31	3.69
*NXNL2*		3.88	4.58	2.83
*OLFML3*		12.78	12.67	6.27
*PDGFRL*	peak1	23.00	28.63	9.84
	peak2	53.61	83.65	21.31
	peak3	12.82	20.11	5.74
	peak4	3.88	6.43	1.43
*PIPOX*	peak1	9.71	11.26	2.87
	peak2	6.57	9.28	3.59
*PKDCC*		11.36	13.27	9.19
*PLAT*		8.55	11.26	3.28
*PPP1R1A*		8.10	6.84	2.40
*RASGRP1*	peak1	0.78	1.11	0.79
	peak2	6.41	7.39	3.74
*RBBP8*	peak1	10.92	13.63	7.07
	peak2	10.10	9.92	3.82
*RIMS4*		21.75	32.17	8.74
*RUBCNL*		34.96	26.54	15.57
*SEZ6*		9.71	11.26	2.87
*SLC19A2*		5.33	7.01	1.99
*SLC7A2*	peak1	23.00	28.63	9.84
	peak2	53.61	83.65	21.31
	peak3	12.82	20.11	5.74
*SLC7A5*		5.44	6.26	1.91
*SLITRK4*		10.88	10.72	3.82

**Table 3:** Central hub genes in the protein–protein interaction (PPI) network derived from the 109 identified genes. Node degree indicates the number of direct connections to neighboring nodes, while betweenness centrality quantifies the extent to which a node resides on the shortest paths between other nodes in the network.

Name	Degree	Betweenness
*NOL6*	81	24,414.77
*FOS*	52	53,050.56
*NOP2*	45	9719.868
*E2F1*	41	32,080.46
*DKC1*	40	38,423.94
*CDC6*	32	17,810
*BMAL1*	26	9105.261
*RBBP8*	23	8415
*E2F6*	18	5803.222
*FKBP4*	17	42,294
*CISH*	14	5031
*AGT*	12	4268
*MYBBP1A*	12	2226.13

## Data Availability

The data presented in this study are openly available in the Gene Expression Omnibus (GEO) database (https://www.ncbi.nlm.nih.gov/geo/) under accession number GSE342249.

## Supplementary Materials

The following supporting information can be downloaded at https://www.mdpi.com/article/10.3390/biom16101392/s1. Figure S1: Analysis of *ACTRT3* gene induction in response to E2, ZEA, and BPA exposure by RT-qPCR. Gene expression changes were assessed 6 h after treatment and are presented as fold changes relative to the vehicle-treated control, calculated from four independent experiments. Statistical significance was assessed using ΔCt values. n.s.: non-significant (one-way ANOVA followed by Dunnett’s multiple comparisons test); Table S1: Sample quality metrics including total reads, mapped reads, FRiP scores, signal-to-noise ratios, duplication rates, Q30 scores, and median peak widths; Table S2: List of consensus peak sets according to the genome-wide peak calling in the four conditions (control, E2-, ZEA-, and BPA-treated). Only peaks identified in both replicates are included. The chromosomal positions of all peaks are reported; Table S3: Comparative peak analysis of cells treated with vehicle (control), E2, ZEA, and BPA. Comparative peak analysis across the different treatments identified seven categories: peaks shared by all treatments; peaks shared between E2 and ZEA, E2 and BPA, or ZEA and BPA; and treatment-specific peaks detected exclusively in E2-, ZEA-, or BPA-treated samples. The chromosomal positions and signal intensities (RPKM values) of all peaks are reported; Table S4: Integration of ChIP-seq and RNA-seq analysis. Integration of ChIP-seq and RNA-seq datasets identified ERα-binding regions located within ±50 kb of the transcription start sites (TSSs) of genes upregulated in response to E2, ZEA, or BPA treatment. A total of 162 ERα-binding regions were associated with 109 upregulated genes. The chromosomal positions of ERα-binding sites and the corresponding gene TSSs are provided. Notably, 17 ERα-binding regions were associated with two genes.

## Abbreviations

The following abbreviations are used in this manuscript:

BPA	Bisphenol A
ChIP-seq	Chromatin immunoprecipitation followed by sequencing
DBD	DNA-binding domain
DCC-FBS	Dextran-coated charcoal-treated fetal bovine serum
E2	17β-Estradiol
ECACC	European Collection of Authenticated Cell Cultures
ERE	Estrogen response element
ERs	Estrogen receptors
FBS	Fetal bovine serum
FC	Fold change
HOMER	Hypergeometric Optimization of Motif EnRichment
IGV	Integrative Genomics Viewer
LBD	Ligand-binding domain
MACS2	Model-based analysis of ChIP-seq 2
MPP	Methyl-piperidinopyrazole
PPI	Protein–protein Interaction
RNA-seq	RNA sequencing
RD	Read distribution
RPKM	Reads per kilobase per million mapped reads
RT-qPCR	Reverse transcription quantitative PCR
TSS	Transcription start site
ZEA	Zearalenone
